# Prevalence of multiple sclerosis in the Lazio region, Italy: use of an algorithm based on health information systems

**DOI:** 10.1007/s00415-016-8049-8

**Published:** 2016-02-17

**Authors:** Anna Maria Bargagli, Paola Colais, Nera Agabiti, Flavia Mayer, Fabio Buttari, Diego Centonze, Marta Di Folco, Graziella Filippini, Ada Francia, Simonetta Galgani, Claudio Gasperini, Manuela Giuliani, Massimiliano Mirabella, Viviana Nociti, Carlo Pozzilli, Marina Davoli

**Affiliations:** Department of Epidemiology, Lazio Regional Health Service, Via Cristoforo Colombo 112, 00142 Rome, Italy; MS Clinical and Research Center, Tor Vergata University and Hospital, Via Montpellier 1, 00133 Rome, Italy; IRCCS Istituto Neurologico Mediterraneo (INM) Neuromed, Via Atinense 18, 86077 Pozzilli, Isernia Italy; Department of Neurology and Psychiatry, Sapienza University, Viale Dell’Università 30, 00185 Rome, Italy; Unit of Neuroepidemiology, Fondazione IRCCS Istituto Neurologico Besta, Via Celoria 11, 20133 Milan, Italy; Department of Neurosciences, S Camillo Forlanini Hospital, Circonvallazione Gianicolense 87, 00152 Rome, Italy; Multiple Sclerosis Center, S Andrea Hospital, Sapienza University, Via di Grottarossa 1037, 00189 Rome, Italy; Multiple Sclerosis Center, Fondazione Policlinico Universitario A. Gemelli, Catholic University, Largo Agostino Gemelli 8, 00168 Rome, Italy; Institute of Neurorehabilitation, Don C Gnocchi Foundation, Via Pier Alessandro Paravia 71, 20148 Milan, Italy

**Keywords:** Multiple sclerosis, Prevalence, Epidemiology, Health information systems, Health administrative data

## Abstract

Compared with other areas of the country, very limited data are available on multiple sclerosis (MS) prevalence in Central Italy. We aimed to estimate MS prevalence in the Lazio region and its geographical distribution using regional health information systems (HIS). To identify MS cases we used data from drug prescription, hospital discharge and ticket exemption registries. Crude, age- and gender-specific prevalence estimates on December 31, 2011 were calculated. To compare MS prevalence between different areas within the region, we calculated age- and gender-adjusted prevalence and prevalence ratios using a multivariate Poisson regression model. Crude prevalence rate was 130.5/100,000 (95 % CI 127.5–133.5): 89.7/100,000 for males and 167.9/100,000 for females. The overall prevalence rate standardized to the European Standard Population was 119.6/100,000 (95 % CI 116.8–122.4). We observed significant differences in MS prevalence within the region, with estimates ranging from 96.3 (95 % CI 86.4–107.3) for Latina to 169.6 (95 % CI 147.6–194.9) for Rieti. Most districts close to the coast showed lower prevalence estimates compared to those situated in the eastern mountainous area of the region. In conclusion, this study produced a MS prevalence estimate at regional level using population-based health administrative databases. Our results showed the Lazio region is a high-risk area for MS, although with an uneven geographical distribution. While some limitations must be considered including possible prevalence underestimation, HIS represent a valuable source of information to measure the burden of SM, useful for epidemiological surveillance and healthcare planning.

## Introduction

Multiple sclerosis (MS) is the most common disabling neurological disorder of young adults around the world. It is typically diagnosed between the ages of 20 and 40, thus affecting individuals in their most productive years. The management of MS is complex and involves a comprehensive team as well as specific diagnostic, therapeutic and rehabilitative services representing a considerable burden for both patients and healthcare systems [1].

Italy is considered a high prevalence country for MS with about 70,000 people affected and an estimated prevalence of approximately 110 per 100,000 population [2, 3]. A number of studies have been conducted in different Italian cities and provinces, mainly based on data of patients recruited in neurological/rehabilitation clinics or MS centers, showing an uneven distribution of MS within the country [4]. It is well known that Sardinia and Sicily (the two largest Italian islands) represent very high-risk areas [5, 6] although a prevalence higher than 100/100,000 has been estimated in some North Italian cities such as Verona, Padova, Ferrara and Genoa [7–10]. While the Northern part of the country as well as Sardinia and Sicily have been particularly well studied, relatively limited epidemiological data are available for Central and Southern regions [11–15]. Moreover, most studies have been carried out at municipal/provincial level while to date prevalence estimates at larger geographic areas (i.e. region) are not common in Italy.

The availability of MS prevalence estimates is considered a valuable information to assess resource utilization and costs associated with the disease as well as for health care planning and monitoring purposes; furthermore, comparing prevalence estimates across different geographical areas can help to understand the contribution of genetic and environmental factors in MS etiology [16].

Different methodological approaches and data sources are used for estimating the prevalence of MS, including case ascertainment from hospitals and clinic records, neurologists, general practitioners (GP) and other physicians, disease registries, and health administrative databases. In recent years, there has been a growing interest in developing methods based on health administrative databases to estimate MS prevalence and different algorithms have been proposed depending on data sources available [17–21].

The Lazio Region is part of the Italian National Health System (NHS) which provides universal health insurance for its residents, including coverage for GP service, hospital services and drug prescriptions. In the Lazio region health information systems (HIS) are comprehensive and contain high-quality information; consequently, health administrative data are largely used to measure the occurrence of acute and chronic diseases [22, 23].

The principal aims of this study were to estimate the prevalence of MS in the Lazio region using population-based health administrative databases and to describe the geographical distribution of MS across Local Health Units (LHUs) and districts within the region. Additionally, we evaluated the validity of the case-finding algorithm based on administrative data using a cohort of patients with definite diagnosis enrolled at MS treatment centers as the gold standard.

## Materials and methods

### Study area

The Lazio region is situated in the central part of Italy; on the west side, it faces for its entire length on the Tyrrhenian Sea and is characterized in the eastern part by a mountainous territory shared with other three regions. Lazio is the third most populated region of Italy and comprises Rome, capital and largest Italian city. The region covers an area of 17,236 km^2^ and includes five provinces and 378 municipalities (among these is Rome). The population on the prevalence day, December 31, 2011, was 5,500,022 inhabitants (2,635,689 males and 2,864,333 females) [24]. Lazio is divided into 12 LHUs representing autonomous bodies of the NHS and a total of 56 health districts nested within them. LHUs are responsible for healthcare organization in specific and homogenous geographical areas in order to provide services in the community closer to where people live.

### Data sources

#### Health administrative data

Data from different regional HIS were used to identify people affected by multiple sclerosis. The Hospital Discharge Registry (HDR) routinely collects data from all regional hospitals, including information on patients’ demographic characteristics, discharge diagnoses and procedures codes according to the International Classification of Disease, Ninth Revision, Clinical Modification (ICD-9-CM). The PHARMED database contains individual records for each drug prescription dispensed by hospital at discharge and by specialist outpatient clinics. Drugs are identified by the national drug register code, which refers to the International Anatomical Therapeutic Chemical Classification System. Individual patient information and drug-dispensing dates are reported for every prescription. In Italy, Disease Modifying Drugs are subsidised by the National Health Service and prescribed, according to the Italian Medicines Agency regulation, to patients attending outpatient clinics in MS specialist centers. The Regional Mortality Registry (ReNCaM) lists causes of death, codified according to the ICD-9, for all resident deaths in the region. The Ticket Exemption Registry (TER) includes data on all residents who are entitled to co-pay fee exemption for some particular conditions, e.g. disability, chronic diseases, low income or old age. As for fee exemption for chronic disease, in Italy very strict rules are in effect within the NHS, entailing patient’s obligation to provide an exhaustive clinical documentation, including definite diagnosis provided by a specialized physician. Finally, the Regional Health Assistance File comprises all resident individuals registered with the Regional Health Service.

#### Clinical data

We used a cohort of MS patients recruited from five specialized high-volume centers to evaluate the validity of the algorithm based on health administrative data. Socio-demographic and clinical data of patients residing in the Lazio region entering the centers in the period 2006–2011 were extracted from medical charts according to a common protocol and entered through a customized software.

### Case ascertainment

We used the HDR, the PHARMED and the TER to identify MS cases in the period from 01/01/2006 to 31/12/2011. From the HDR we selected all patients who were hospitalized in the Lazio Region with a primary or a secondary diagnosis of MS (ICD9-CM: 340.0); from the PHARMED we selected all patients with at least one pharmacy claim in the study period for at least one among Interferon beta-1a (Avonex, Rebif), Interferon beta-1b (Betaferon, Extavia), Copaxone (Glatiramer Acetate) and Tysabri (Natalizumab); finally, from the TER we selected all patients registered with the MS specific code (046.340). Each patient was selected at the date of the first claim recorded in the regional information systems. All residents of Lazio region served by the public health service are uniquely assigned a personal identification number recorded in all the regional healthcare databases. This individual identifier provides the key to link different databases and allows the identification of individuals and avoid double counting. We excluded from the analysis patients who died during the study period identified through a record linkage with the ReNCaM and patients not residing and not registered on the prevalence day with the Lazio Region Health Service, traced using the Health Assistance File.

### Statistical analysis

#### Prevalence

The prevalence day was December 31, 2011. Age- and gender-specific prevalence rates per 100,000 were calculated using the number of MS patients that were alive on the prevalence day as numerator and the population of residents in the Lazio region the same day as denominator. Standardized prevalence rates were also calculated by the direct method separately for males and females using the European Standard Population for reference.

To explore the geographic pattern of MS prevalence within the region, standardized prevalence ratios were calculated for each of the 12 LHUs and 56 districts. To remove the possible bias effect due to different age and gender structures for different geographic areas, age and gender-standardized prevalence rates with 95 % confidence intervals (CI) were calculated by the direct method. We used a multivariate Poisson regression with no intercept and centered covariates, in which in addition to gender and age, were included *n* dummy variables that represent the areas being compared. The parameters estimated by the model were used to calculate the expected rates if all the considered areas had the same distribution of the general population by age and gender. The expected rate obtained for each areas were subsequently corrected by a multiplicative factor *k* which takes into account the non linear nature of the model applied.

$$K = {\text{ actual number of events}}/\sum\limits_{j = 1}^{m} {p_{j} \times n_{j} }$$

where *p*_*j*_ is the expected rate, *n*_*j*_ is the group size, and *m* is the number of groups.

Prevalence ratios (PR) were estimated to assess the excess risk in each LHU and district compared with the regional estimate. Patients’ residential addresses were used to analyze MS geographic distribution within the region. All statistical analyses were conducted using SAS, version 9.2.

Furthermore, maps of standardized prevalence rates were produced to compare the prevalence of MS in each LHU and district (ArcGIS 9.2 software). The classes used in the maps have been calculated applying the Jenks natural breaks optimization algorithm, which reduces the variance within classes and maximizes the variance between classes [25].

#### Validity of the case ascertainment algorithm

Data of MS patients recruited at the treatment centers were linked to prevalent cases on December 31, 2011 identified through HIS. Considering clinical data as the gold standard, we calculated the proportion of individuals correctly identified as MS cases.

## Results

### Prevalence

**Fig. 1:**
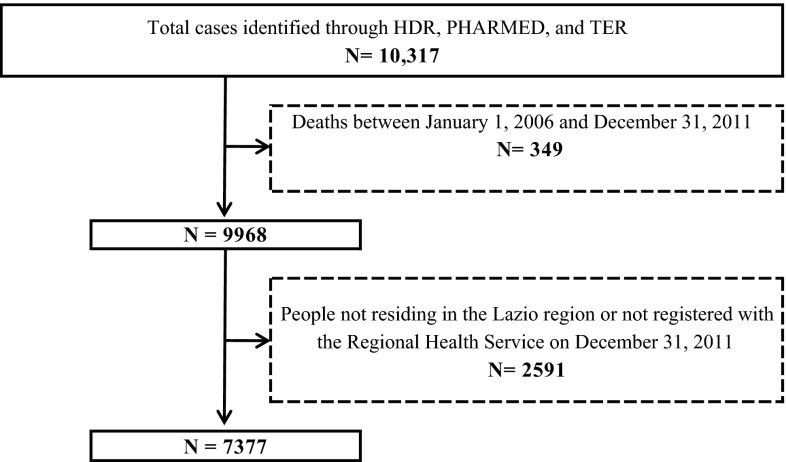
Flow chart of MS cases selection from regional health information systems

**Fig. 2:**
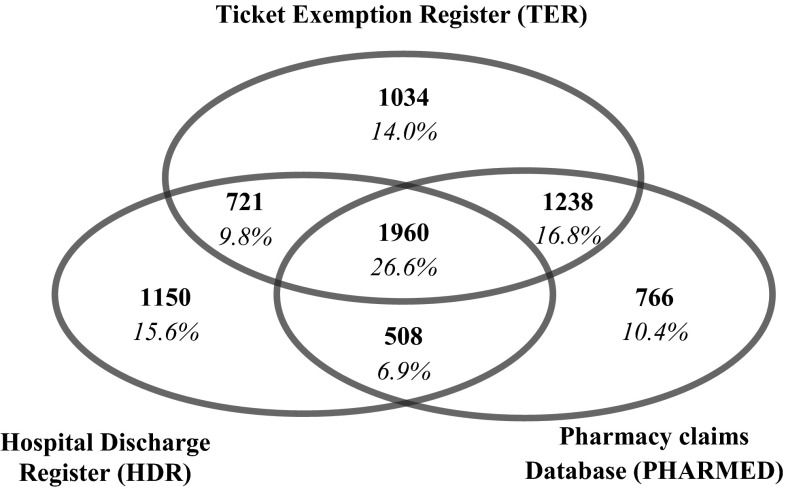
Contribution of regional health information systems to MS cases identification

Through the regional HIS we identified a total of 7377 MS patients residing in the Lazio region on the prevalence day, 2427 males and 4950 females (Fig. 1). Figure 2 shows the contribution of different data sources to the identification of MS cases: 1150 (15.6 %) patients were present exclusively in the HDR, and among these 329 (28.6 %) had more than one hospitalization during the study period; 1034 (14.0 %) were found in the TER only and 766 (10.4 %) in the PHARMED (599, 78.2 %, with more than one prescription claim in the study period). Finally, about 27 % of cases was registered in all the databases.

At prevalence day, mean age was 45.9 years (SD 12.9) without differences between genders. The crude regional prevalence was 130.5/100,000 (89.7/100,000 for males and 167.9/100,000 for females) with a male to female ratio of 1:1.9. Prevalence estimate standardized to the European Standard Population was 119.6/100,000 (95 % CI 116.8–122.4), 81.4/100,000 and 155.5/100,000 for males and females, respectively.

Age- and gender-specific prevalence estimates are reported in Table 1. The prevalence peaked in the age groups 35–44 and 45–54 for both males and females. Table 2 shows crude and standardized prevalence and prevalence ratios by LHU. The standardized prevalence ranged from 96.3 (95 % CI 86.4–107.3) for Latina to 169.6 (95 % CI 147.6–194.9) for Rieti with statistically significant variations among LHUs.

**Table 1:** Age- and gender-specific prevalence per 100,000 population on December 31, 2011, Lazio region, Italy

Age classes	Female					Male					Total				
	Cases	Population^a^	Prevalence	95 % CI		Cases	Population^a^	Prevalence	95 % CI		Cases	Population^a^	Prevalence	95 % CI	
				Inf.	Sup.				Inf.	Sup.				Inf.	Sup.
0–14	10	377,117	2.7	1.0	4.3	8	400,174	2.0	0.6	3.4	18	777,291	2.3	1.2	3.4
15–24	161	258,499	62.3	52.7	71.9	86	272,381	31.6	24.9	38.2	247	530,880	46.5	40.7	52.3
25–34	786	337,250	233.1	216.8	249.3	386	327,865	117.7	106.0	129.5	1172	665,115	176.2	166.1	186.3
35–44	1444	480,267	300.7	285.2	316.2	687	451,034	152.3	140.9	163.7	2131	931,301	228.8	219.1	238.5
45–54	1335	458,242	291.3	275.7	306.9	628	429,264	146.3	134.9	157.7	1963	887,506	221.2	211.4	231.0
55–64	814	370,602	219.6	204.6	234.7	400	337,570	118.5	106.9	130.1	1214	708,172	171.4	161.8	181.1
65+	400	665,683	60.1	54.2	66.0	232	487,060	47.6	41.5	53.8	632	1,152,743	54.8	50.6	59.1
TOT	4950	2,947,660	167.9	163.3	172.6	2427	2,705,348	89.7	86.1	93.3	7377	5,653,008	130.5	127.5	133.5
Standardized rate^b^			155.5	151.1	160.0			81.4	78.1	84.7			119.6	116.8	122.4

^a^Population of residents in the Lazio region and alive on the prevalence day (December 31, 2011)

^b^Prevalence × 100,000 standardized to the European Standard Population

**Table 2:** Crude and standardized prevalence and prevalence ratios (PR) by Local Health Unit per 100,000 population, Lazio region, Italy

Local health unit	Cases	Crude prevalence	Standardized prevalence	95 % CI	PR	*p* value
RM A	742	137.3	134.0	121.4–147.9	1.03	0.604
RM B	1008	147.5	147.5	134.6–161.6	1.13	0.009
RM C	783	147.4	147.6	133.9–162.6	1.13	0.013
RM D	714	129.2	129.5	117.3–143.1	0.99	0.885
RM E	704	140.7	140.8	127.4–155.6	1.08	0.136
RM F	349	114.1	112.9	99.7–127.9	0.87	0.023
RM G	658	136.9	136.8	123.6–151.5	1.05	0.363
RM H	599	110.5	110.0	99.1–122.1	0.84	0.001
Viterbo	345	111.0	112.1	98.9–127.1	0.86	0.017
Rieti	260	165.5	169.6	147.6–194.9	1.30	0.000
Latina	533	95.9	96.3	86.4–107.3	0.74	0.000
Frosinone	682	138.3	140.5	127.0–155.4	1.08	0.151
Lazio region	7377	130.50				

**Fig. 3:**
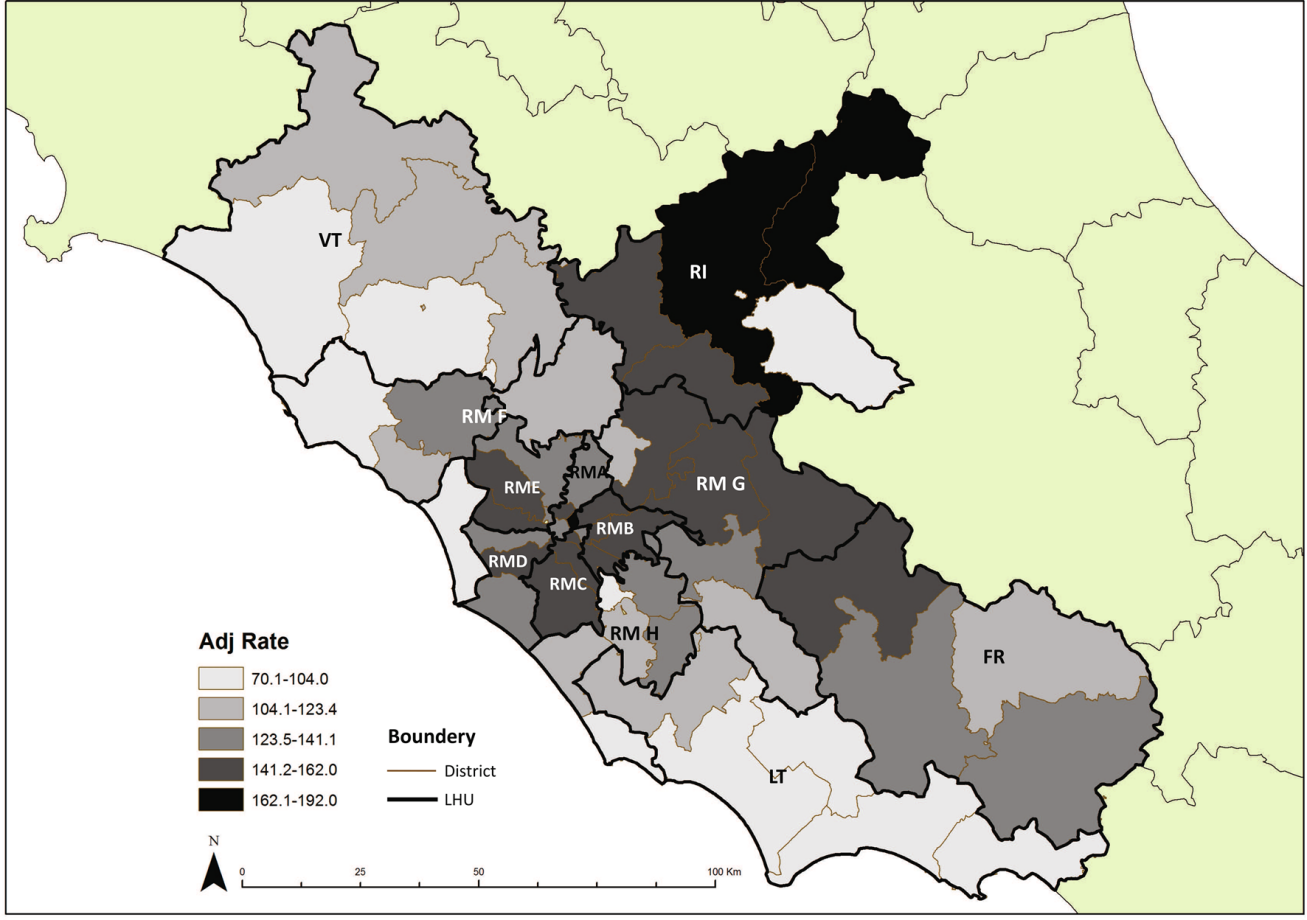
Age- and gender-adjusted MS prevalence rates, per 100,000 population by district in the Lazio region, Italy

Compared with the regional average, MS prevalence was significantly higher in Rieti (PR = 1.30) and to less extent in two LHUs within Rome (RM B and RM C, PR = 1.13) while lower prevalence was observed in Latina (PR = 0.74), Viterbo (PR = 0.86) and one LHU in the province of Rome (RM H, PR = 0.84). Distribution of MS showed a large variation between districts even within the same LHU (Fig. 3). Most of districts located in the eastern part of the region had relatively high prevalence, in particular within the LHU of Rieti, RM G and Frosinone, with estimates included between 141/100,000 (95 % CI 115.4–172.3) and 192/100,000 (95 % CI 160.9–229.1). On the contrary, in the coastal area of the region low prevalence estimates were observed, in particular in the LHU of Latina where prevalence ranged between 70/100,000 (95 % CI 50.6–97.0) and 96/100,000 (95 % CI 78.2–118.1).

### Validity of the case ascertainment algorithm

Among 3881 patients enrolled at the specialist centers during the study period, 3297 (85 %) were included in the population of cases identified through regional HIS. Among those not found (*N* = 584), there were 171 cases for which the personal individual number identifying each subject receiving services within the Regional Health Service, was not available. The remaining 413 patients, although having the unique identification code, were not captured by our algorithm.

## Discussion

This study contributes to the knowledge on the epidemiology of MS in Southern Europe. In particular, it provides new insights on MS prevalence in Central Italy, an area that has been considerably less studied than Sicily and Sardinia and the Northern part of the country. We used health information systems to produce a prevalence estimate of MS at the regional level and to describe its geographic distribution across different areas within the region. The large population enabled us to provide a robust estimate of MS prevalence which ranks Lazio between the areas at high risk in Italy, although with an uneven distribution across LHUs and districts.

A huge number of studies assessed MS prevalence in European countries, revealing spatial heterogeneity in the distribution of disease [2, 26]. Italy has been particularly well studied, although most of studies were carried out in the Northern areas of the country and in the two largest islands. Considering prevalence estimates from 2000 onwards, they ranged from 94/100,000 to 149/100,000 in the North [27, 28], and from 127/100,000 to 224/100,000 in the islands [6, 29]. Moreover, some studies revealed an heterogeneous distribution of the disease even within small areas [5, 27], calling for specific analytical studies to detect possible MS risk factors.

So far only few data were available on MS prevalence in Central Italy coming from studies conducted in the district of L’Aquila and in some areas of the Lazio region [11–13]. In a 2007 prevalence study conducted in the province of Frosinone the overall crude prevalence was 95.0 per 100,000 (94.4 when standardized on the European population) [12]. Applying capture–recapture techniques a period prevalence of 41.3/100,000 was estimated in the city of Rome between 1980 and 1990 [13]. Both in Frosinone and Rome we observed higher prevalence estimates than those previously reported. Although comparing results across studies presents a number of challenges, this finding is consistent with results of several studies showing a progressive increase in MS prevalence in different Italian cities and provinces [8, 28] as well in other countries [30].

Different methods for calculating MS prevalence based on health administrative data have been recently developed [17–21] demonstrating the growing interest in the use of HIS as a source of information to estimate and monitor the burden of the disease. Administrative databases are similar among studies although algorithms may vary according to different context or specific objectives [19].

Given the good quality and completeness of health administrative databases in the Lazio region, they have been increasingly used to estimate the prevalence of chronic diseases and to measure the performance of health care organizations and the quality of services offered [22, 23, 31]. To identify cases of MS we used hospital discharge diagnoses, prescriptions of medications used exclusively for the treatment of MS and disease-specific ticket exemption code. The recently proposed algorithms for identifying MS cases from administrative databases mainly rely on drug prescriptions, in particular in those countries, like Italy, with a universal health coverage [20, 21]. Considering the high cost of drugs for the treatment of MS, the self-funding of these medications is likely to be negligible. It is known that some patients with MS do not take drugs or use drugs other than those included in the algorithm and therefore they cannot be captured through medication claims. The use of both hospital discharge and exemption registries allowed us to recover part of this untreated or differently treated population. In fact, although there are large overlaps between the populations registered in the three databases, each source of data provides a specific and unique contribution to the identification of MS cases. It is clear that the algorithm based on administrative records only allows to identify patients who have contacts with the Regional Health Service that, considering the characteristics of the disease and its chronic nature, is supposed to be a very likely event. Nonetheless, the overall prevalence of MS may be underestimated by the approach we used. As part of this study, data from medical charts of patients entering five MS centers in the period 2006–2011 were collected. By record linkage procedures, we estimated that 85 % of patients attending the centers were included in the population identified by the algorithm based on health administrative data. While this figure is not far from sensitivity estimates observed in validation studies in other settings [22], it is likely that patients in the first steps of the disease with a clinical examination in an ambulatory specialist setting and/or untreated are not detected through our methodology. Another reason for the lack of complete identification of clinical records in our administrative datasets may be in the low quality in reported personal demographic data in the medical charts with consequent failed record linkage procedures. On the other hand, the population of MS patients identified showed a male to female ratio and an age distribution consistent with those reported by other studies carried out both in Italy and elsewhere supporting the reliability of our results [5, 6, 9, 11].

The study also showed an uneven geographical distribution, with a gradient in prevalence decreasing from the eastern mountainous part of the region to the western areas closer to the coast. This is in line with other studies conducted at different latitudes, showing lower MS incidence in the coastal area compared to the inland area [32, 33]. Diet and lifestyle factors have been hypothesized to modulate the risk of MS across regions at the similar latitude. Lifestyles of people in coastal areas, generally involving more time outdoors, match evidence showing that exposure to sunlight is associated with a lower risk of developing MS [34–36]. As sun exposure is the determining factor for vitamin D status in most populations, it has been suggested that sun exposure to some extent mediates its effect on MS risk through vitamin D [37].

This study has both strengths and limitations. The standardized methodology and the contribution of medical records in validating the case identification algorithm are the main strengths of the study. Our procedure based on population-based administrative data has different advantages: it is time efficient, allows to easily provide updated MS estimates, and enables to avoid problems related to small sample size. We chose to ascertain an MS case from at least one claim in one of the three administrative databases due to MS clinical practices and the reliable coding process utilized within the Lazio region.

The major limitation is the probable underestimation of MS prevalence. Although the use of a combination of multiple sources of data (hospital discharge, ticket exemption, and prescription registries) contributes to produce more reliable estimates, only cases diagnosed and recorded in administrative databases could be captured by the algorithm. The availability of a cohort of patients with definite diagnosis of MS allowed us to calculate the proportion of cases not identified through HIS and therefore to estimate the extent of the actual population prevalence underestimation. Secondly, the use of administrative data instead of clinical information to identify the population affected by MS limits its use for more analytical purposes.

In conclusion, this study produced an estimate of MS prevalence in the Lazio region using population-based health administrative databases and described the geographical distribution of MS within the region. Although some limitations must be considered including possible prevalence underestimation, administrative databases represent an attractive source of information to measure the burden of MS allowing for periodic updates of prevalence estimates, useful for monitoring prevalence trends at population level and to ensure appropriate healthcare resources allocation. Moreover, considering that we used administrative databases including information available in other Italian regions, the proposed algorithm could be tested to obtain population-based prevalence estimates of MS in different areas of the country and to analyze geographic differences as in the case of other chronic diseases [38].
